# The air quality impacts of road closures associated with the 2004 Democratic National Convention in Boston

**DOI:** 10.1186/1476-069X-5-16

**Published:** 2006-05-26

**Authors:** Jonathan I Levy, Lisa K Baxter, Jane E Clougherty

**Affiliations:** 1 Department of Environmental Health, Harvard School of Public Health, Landmark Center 4^th ^Floor West, P.O. Box 15677, Boston, MA, 02215, USA

## Abstract

**Background:**

The Democratic National Convention (DNC) in Boston, Massachusetts in 2004 provided an opportunity to evaluate the impacts of a localized and short-term but potentially significant change in traffic patterns on air quality, and to determine the optimal monitoring approach to address events of this nature. It was anticipated that the road closures associated with the DNC would both influence the overall air pollution level and the distribution of concentrations across the city, through shifts in traffic patterns.

**Methods:**

To capture these effects, we placed passive nitrogen dioxide badges at 40 sites around metropolitan Boston before, during, and after the DNC, with the goal of capturing the array of hypothesized impacts. In addition, we continuously measured elemental carbon at three sites, and gathered continuous air pollution data from US EPA fixed-site monitors and traffic count data from the Massachusetts Highway Department.

**Results:**

There were significant reductions in traffic volume on the highway with closures north of Boston, with relatively little change along other highways, indicating a more isolated traffic reduction rather than an across-the-board decrease. For our nitrogen dioxide samples, while there was a relatively small change in mean concentrations, there was significant heterogeneity across sites, which corresponded with our *a priori *classifications of road segments. The median ratio of nitrogen dioxide concentrations during the DNC relative to non-DNC sampling periods was 0.58 at sites with hypothesized traffic reductions, versus 0.88 for sites with no changes hypothesized and 1.15 for sites with hypothesized traffic increases. Continuous monitors measured slightly lower concentrations of elemental carbon and nitrogen dioxide during road closure periods at monitors proximate to closed highway segments, but not for PM_2.5 _or further from major highways.

**Conclusion:**

We conclude that there was a small but measurable influence of DNC-related road closures on air quality patterns in the Boston area, and that a low-cost monitoring study combining passive badges for spatial heterogeneity and continuous monitors for temporal heterogeneity can provide useful insight for community air quality assessments.

## Background

Occasionally, "natural experiments" occur that provide the opportunity to evaluate the influence of changes in emissions on changes in ambient concentrations and/or health effects. For example, the closing and reopening of a steel mill in Utah Valley was associated with changes in PM_10 _concentrations and respiratory hospital admissions in the winter [1]. A coal ban in Dublin resulted in significantly (70%) lower black smoke levels and corresponding reductions in cardiopulmonary mortality [2], and a widespread electricity blackout in the summer of 2003 in multiple North American cities yielded 90% reductions in sulfur dioxide (SO_2_) concentrations, 50% reductions in ozone concentrations, and 70% reductions in particle light scattering relative to time periods of normal operation [3].

More common "natural experiments" involve substantial changes in traffic patterns. Major sporting events, holidays, and road construction can all influence traffic volume on selected roadways, although less transient changes that can be anticipated in advance are less common. In one such example, reductions in traffic volume associated with the 1996 Summer Olympics in Atlanta were associated with significant improvements in ambient air quality and reductions in childhood asthma events [4]. In addition, construction of a bypass in North Wales was shown to reduce heavy goods traffic and particulate matter concentrations by 23–29%, with some concurrent reductions in rhinitis and rhinoconjunctivitis [5].

These studies were able to capture the temporal trend in concentrations with a limited number of monitors, given relatively large emissions changes and the emphasis on either regional air pollutants [4] or small geographic areas [5]. However, it is often of policy relevance to evaluate impacts of local air pollutants over a broad geographic area, as many policies (i.e., the congestion charge in London) have impacts on numerous roads, including shifting traffic from one road to another.

The Democratic National Convention (DNC) in Boston in 2004 (July 26–29) provided an opportunity to evaluate the impacts of short-term but potentially significant changes in traffic patterns on air quality. In March 2004, the U.S. Secret Service announced numerous road closures as a homeland security measure [6], which were extensively publicized for the weeks and months leading up to the convention [7-9].

Road closures of this magnitude would be expected to have a few major impacts. Some drivers will take alternate routes, leading to traffic increases on those routes relative to normal traffic volume. Some commuters will shift their work schedules to avoid travelling during the times of the road closures, potentially leading to a shift in the timing of congestion and associated emissions. However, the most significant effect given the extensive media coverage and concerns about massive congestion may involve drivers avoiding commuting altogether (i.e., working from home, leaving town, or shifting to public transportation). Regardless of the direction of the net effect, it was anticipated that the road closures associated with the DNC could have a measurable influence on the geographic pattern of traffic-related air pollution as well as the overall concentrations.

The pollutants of interest for mobile sources include multiple primary pollutants, for which the concentration gradient is such that monitors not proximate to the roadway may not detect the impacts of emission changes. For example, nitrogen dioxide (NO_2_) concentrations display significant between-site variation in urban settings [10], with the heterogeneity linked to traffic type and volume as well as land use patterns [11]. Although PM_2.5 _concentrations generally display greater temporal than spatial heterogeneity [12-15], given a substantial contribution of regional sources, models have demonstrated a significant influence of traffic intensity, road characteristics, and proximity to roadways on PM_2.5 _concentrations [16]. Furthermore, the particle size distribution and number concentration changes significantly within tens of meters of a roadway [17,18], and elemental carbon (EC) concentrations are significantly higher near major roads [13,19]. Thus, central-site monitors may not capture the effects of localized changes in traffic patterns and/or emissions, implying that a different monitoring strategy may be necessary.

Determining the air quality impacts of the DNC requires a monitoring strategy with sufficient spatial coverage to capture distributional effects and sufficient temporal resolution to evaluate road closures during only a portion of the day for only a few days. In any study of this sort, there is an inherent trade-off between using continuous and integrated samplers. Continuous samplers have the distinct advantage of being able to evaluate diurnal patterns that may correspond with changes in traffic flow, but are usually expensive and resource-intensive, making it difficult to take measurements at many sites at once and to evaluate spatial patterns. On the other hand, integrated samplers often correspond more closely with Federal Reference Methods and can be deployed at many more sites (especially if passive samplers are used), but by their nature they cannot capture diurnal patterns and may lack the power to detect incremental emissions changes.

Thus, we have two primary aims in this analysis. We wish to determine the influence of the road closures associated with the Democratic National Convention on the magnitude and distribution of traffic-related air pollution in the Boston metropolitan area. More generally, we aim to evaluate the strengths and weaknesses of alternative monitoring strategies to capture the air quality impacts of traffic changes, and specifically to determine if inexpensive, short-term monitoring campaigns can provide useful insight in the event of natural experiments or other short-term local emissions changes. A monitoring protocol that could capture the effects of these road closures, given both financial and technical constraints, could have numerous applications for community air quality studies and epidemiological investigations.

## Methods

### Event characteristics

**Figure 1:**
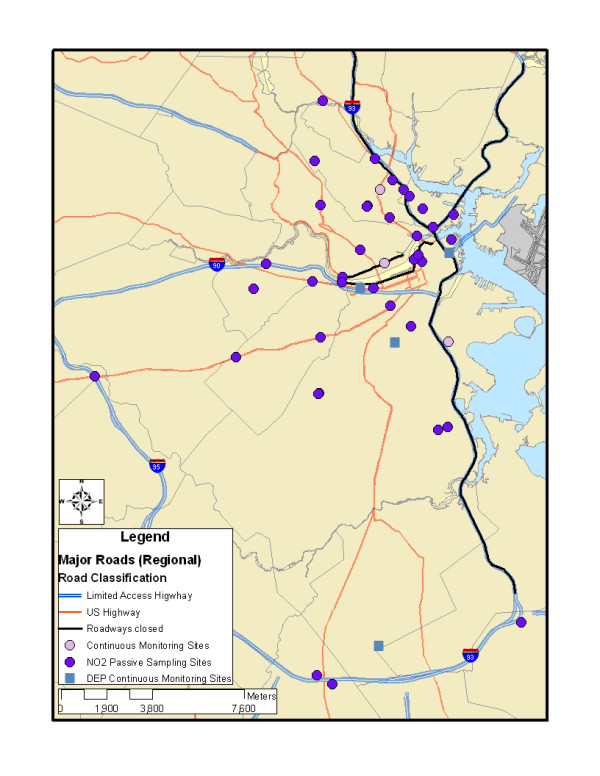
Air pollution sampling sites and road closures associated with the 2004 Democratic National Convention.

The Democratic National Convention was held at the FleetCenter (now TD Banknorth Garden) in Boston from July 26–29, 2004. As the FleetCenter is proximate to multiple major highways, road closures were proposed to limit traffic on those roadways, as well as to reduce the possibility of resulting traffic jams elsewhere. Approximately 40 miles of roads were closed for some period of time (generally from 4 pm to midnight), including the major north-south highway into Boston (I-93) and multiple surface and feeder roads (Figure 1). These road closures were publicized in order to reduce traffic volumes, with the explicitly stated goal to reduce traffic volumes on I-93 by at least 50% [20].

### Sampling site selection

We selected monitoring sites intended to capture the array of hypothesized impacts from the DNC. Based on the planned road closures (Figure 1), we identified highway or road segments that could potentially be influenced in different ways by the changes in traffic volume. We sampled at four different categories of sites:

1) Sites with hypothesized concentration decreases: Proximate to a closed-down road or highway but not proximate to an identified alternate route.

2) Sites with hypothesized concentration increases: Proximate to an identified alternate route but not a closed-down road.

3) Sites with no change hypothesized: Geographically isolated from the road closures or alternate routes.

4) Site with unclear impacts *a priori*: Site with multiple countervailing influences. For example, measurements taken near a highway without road closures could have concentration decreases if overall traffic were reduced, but could have concentration increases if these roads were used as alternate routes to downtown Boston.

We selected 40 sites across the Boston metropolitan area with approximately equal representation in these four categories. The sample size of 40 was chosen partly due to logistical feasibility, but some studies have found this approximate sample size to have adequate statistical power for capturing traffic-linked concentration heterogeneity [16,21-23]. Of note, we selected sites as close to the roadway in question as was feasible, with no major obstructions between the roadway and the monitor, so our ability to detect spatial gradients was enhanced relative to some study designs. Aside from our *a priori *classifications, we also selected sites representative of traffic to the north, south, and west of downtown Boston (which is located in eastern Massachusetts and is bordered by the ocean to the east), to ensure meaningful spatial coverage. In general, as it was unclear precisely which roads would be most influenced by the closures, we aimed to capture the effects of highways, other major arterials, and surface roads near highway exits in a variety of locations. Our sampling sites are presented in Figure 1.

### Sampling methods

Given the strong relationship shown elsewhere between traffic volume and NO_2 _concentrations, as well as the availability of inexpensive passive samplers, we considered NO_2 _as our primary measurement to address spatial heterogeneity in concentrations. While more near-source heterogeneity may be observed for NO, the steep drop-off in concentrations would lead to detection difficulties where monitors were not immediately proximate to the roadway (i.e., highways). NO_2 _concentrations were measured with Yanagisawa passive filter badges, which absorb NO_2 _on a triethanolamine solution on a cellulose fiber filter [24]. Each badge was exposed for seven days duration to assure concentrations above the limit of detection. Badges were swapped weekly at each site, providing samples corresponding to the week before, during, and after the DNC. Duplicate samples and field blanks were used at 10% of sites, selected by random number generation.

In addition, at three of these 40 sites, we collected continuous measures of elemental carbon (EC) and/or fine particulate matter (PM_2.5_) (Figure 1). These three sites were selected to capture different geographic areas and three of the hypothesized phenomena associated with the DNC road closures. One site to the north of downtown (Somerville) was located alongside Route 28, an alternate route to the closed I-93 South, where it was hypothesized that traffic impacts would increase during the DNC (Category 2 above). A second site to the west of downtown (Cambridge) was located along Memorial Drive, a road that was scheduled for closure after 4 pm during the DNC (Category 1 above). The third site to the south of downtown (South Boston) was proximate to the closed I-93 but did have potential local traffic contributions given proximity to auxiliary roads that converge at a five-way intersection close to the sampling site (Category 4 above).

At each of the three continuous monitoring sites, we measured EC using a Magee Scientific portable aethalometer (Berkeley, CA), which uses a continuous filtration and optical transmission technique to capture near real-time EC concentrations. Five-minute average concentrations were collected, which were aggregated to one-hour averages using the Aethalometer Data Masher Version 4.2g . In addition, at each site, we collected temperature and relative humidity at 10-minute intervals using the HOBO outdoor sensor and data logger (Onset Computer Corporation, Pocasset, MA).

Additionally, at the Cambridge and South Boston sites, we measured wind speed and direction using the Weather Wizard III (Davis Instruments, Hayward, CA). At the Cambridge site, given the known change in traffic volume immediately proximate to the sampling site, we also measured PM_2.5 _using the DustTrak Model 8520 (TSI, Minneapolis, MN), a laser photometer fitted with an impactor to exclude larger particles. Continuous traffic counts on the side of Memorial Drive closed during the DNC were measured using the Trax I (JAMAR Technologies, Horsham, PA), an automatic traffic data recorder that registers all vehicles passing over pneumatic tubing.

For the weeks before, during, and after the DNC, we also obtained ambient concentration data from all EPA monitors in and around the Boston downtown area. This included hourly average concentrations of PM_2.5 _at an urban central city site (Roxbury), an urban site near I-93 (North End), and a background site south of Boston (Blue Hills). EC concentrations were also available from the Roxbury and North End monitors. Nitrogen dioxide was available from Roxbury as well as an urban high-traffic site (Kenmore Square) and background sites north (Lynn) and south (Blue Hills) of Boston. Ozone concentrations, used to indicate general meteorological trends and possible modifying effects on nitrogen dioxide, were available from Lynn and Roxbury as well as a background monitor near Boston (Long Island). We additionally collected temperature, relative humidity, barometric pressure, and wind speed and direction from the Roxbury monitor, as well as hourly precipitation from the EPA monitor in Lynn. Hourly traffic volumes on major highways surrounding Boston were obtained from the Massachusetts Highway Department. Of note, time-resolved traffic data were available for major highways (I-93, I-95, Route 1), but no such data were available for surface roads, other than from our monitoring on Memorial Drive in Cambridge. All monitoring sites were geocoded using ArcGIS Version 9.0 (ESRI, Redlands, CA) and aerial photography of the region.

### Analytical methods

To determine if the DNC resulted in significant changes in traffic volumes and/or air pollutant concentrations, we evaluated integrated nitrogen dioxide concentrations and traffic volumes by calculating the ratio of the level during the week of the DNC to the average of levels the weeks before and afterwards. Ratios below 1.0 indicate a reduction in traffic volumes or concentrations during the DNC. In addition, we used paired two-sample t-tests to determine whether nitrogen dioxide concentrations were significantly different during the DNC as compared with weeks before or afterwards, either in total or within categories of sampling sites. We further considered whether *a priori *classification significantly predicted concentration ratios.

## Results

### Meteorological conditions

Temperature and humidity profiles were nearly identical using data from the EPA monitoring station in Roxbury and using our HOBO measurements. Mean daily temperatures ranged between 66 and 82 degrees F, with mean daily relative humidity between 65% and 94%. During the week of the DNC, the mean temperature was somewhat lower than the weeks before and after the DNC (71 degrees F vs. 77 degrees F), with comparable humidity. The wind was typically out of the southwest or southeast, although measurements taken in Cambridge (between the Charles River and a series of tall buildings) indicated a stronger northwesterly component. The average wind speed was slightly lower during the week of the DNC (4.0 mph, versus 4.5 mph the week before the DNC and 5.1 mph the week after the DNC). Precipitation was generally low, with only trace rainfall the week prior to the DNC, and significant rainfall on the Saturday prior to the DNC (7/24/04) and on the Thursday following the DNC (8/5/04).

### Traffic volumes

**Figure 2:**
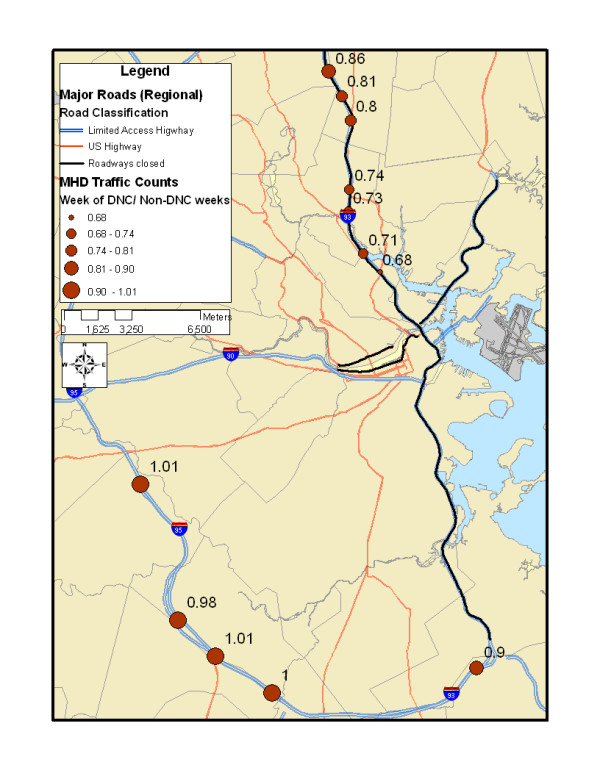
Traffic volume change during the Democratic National Convention, as compared with the identical days of the week during the weeks before and after the convention. Values represent ratio between DNC traffic volume and non-DNC traffic volume.

As indicated in Figure 2, there was a significant reduction in traffic on I-93 north of the city (where road closures occurred), with greater reductions at exits closer to downtown Boston, potentially indicating increased use of auxiliary roads close to the city relative to other weeks. In contrast, there was very little change in traffic on a loop road around Boston (I-95), which lacked road closures, or I-93 to the south or west, indicating a more isolated traffic reduction rather than an across-the-board decrease.

**Figure 3:**
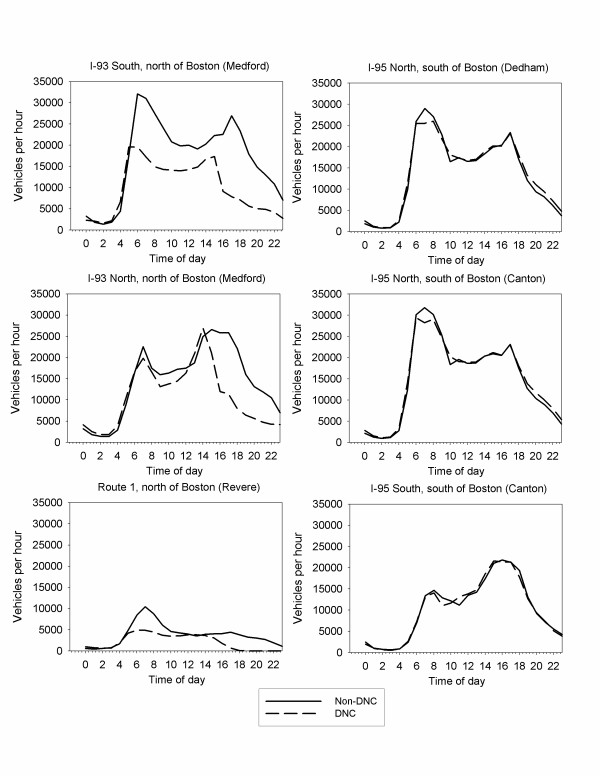
Hourly traffic counts during the DNC (July 26–29) and during corresponding weekdays before and after the DNC, on the major north-south highways with road closures (I-93 and Route 1) and a loop road without road closures (I-95).

A more detailed hourly analysis confirms these observations. Focusing on hourly data for the four days of the DNC (July 26–29) as compared with the identical weekdays the week before and after the DNC, the hourly average traffic counts are essentially unchanged on I-95 (Figure 3). In contrast, there are significant changes on I-93 and Route 1 north of Boston, both of which had road closures associated with the DNC, with the precise diurnal patterns and impacts varying by direction and proximity to downtown Boston.

### Integrated nitrogen dioxide samples

**Figure 4:**
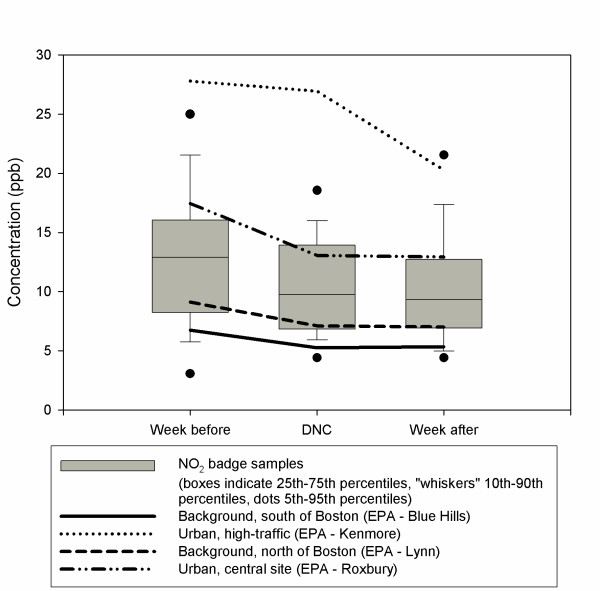
Nitrogen dioxide concentrations the week before, during, and after the Democratic National Convention, as measured at EPA fixed-site monitors and 40 study integrated sampling sites.

Looking across all samples, the mean at our sampling sites was 13 ppb the week before the DNC, 10 ppb the week of the DNC, and 11 ppb the week after the DNC. This general time trend is comparable to the trends detected at most EPA monitors, with the exception of the urban high-traffic monitor (Figure 4). In paired two-sample t-tests, concentrations during the DNC were moderately lower than the average of the weeks before and afterwards (p = 0.10). However, comparisons across all sites only provides limited insight, as there was significant heterogeneity across our sampling sites in both concentrations and in changes associated with the DNC.

**Figure 5:**
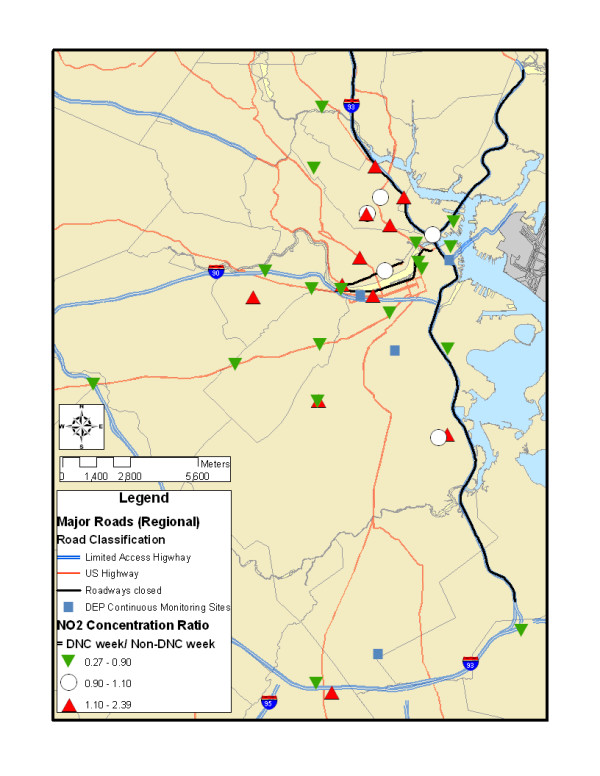
Ratio between nitrogen dioxide concentrations during the DNC and the average of concentrations the weeks before and after the DNC, from the study integrated sampling sites.

For the ratio between DNC and non-DNC concentrations, spatial patterns are mixed, although concentration ratios appear to be lower in proximity to the downtown area and higher near potential exit points from the north-south highway with road closures (Figure 5). When we look at our *a priori *road classifications (Table 1), those sites for which traffic was anticipated to decrease had a median concentration ratio of 0.58, versus median ratios of 0.88 for "no change" sites and 1.15 for sites where traffic was expected to increase. Although our statistical power is limited for stratified analyses, there is evidence that mean concentrations during the DNC were lower at the sites with hypothesized concentration decreases or unclear *a priori *impacts (p = 0.10 and 0.05, respectively), higher at the sites with hypothesized concentration increases (p = 0.13), and unchanged at the sites with no hypothesized change (p = 0.79).

**Table 1:** Nitrogen dioxide concentrations (ppb) during DNC and non-DNC weeks, stratified by *a priori *traffic classification.

Category	Median concentration and range, DNC	Median concentration and range, non-DNC ^1^	Median concentration ratio (DNC/non-DNC) and range
1 – Hypothesized decrease (n = 7)	7 (3 – 16)	10 (6 – 20)	0.58 (0.27 – 2.0)
2 – Hypothesized increase (n = 9)	14 (7 – 19)	12 (3 – 15)	1.15 (0.51 – 1.88)
3 – Hypothesized no change (n = 11)	11 (6 – 19)	12 (7 – 17)	0.88 (0.82 – 1.23)
4 – Unclear impacts *a priori *(n = 7)	7 (6 – 9)	12 (4 – 18)	0.70 (0.38 – 2.4)

^1 ^Average of concentration the week before and after the DNC

Of note, the sites with uncertain *a priori *impacts had the greatest heterogeneity in ratios (highest coefficient of variation), potentially reflecting differences in traffic impacts across the city. For example, many of those sites with uncertain *a priori *impacts that had low concentration ratios were near east-west roads that could have been used as part of alternate routes into Boston. These low ratios, coupled with the lack of traffic volume changes on I-95 (the road that would be taken to reach these alternate routes), indicate that there was little evidence that traffic from I-93 was displaced onto other highways rather than surface roads.

### Continuous monitoring data

**Figure 6:**
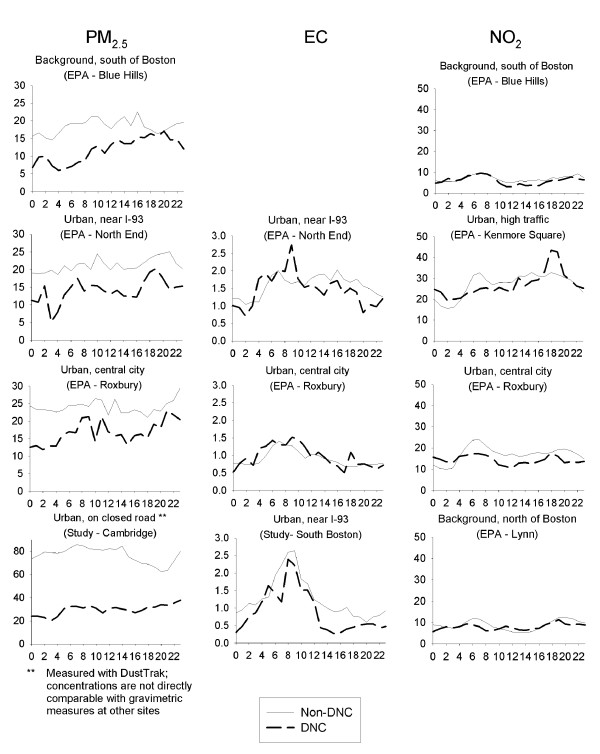
Concentration patterns during the Democratic National Convention (July 26–29) versus concentrations during the same days of the week before and after the Democratic National Convention. x-axis = hour of day; y-axis = concentration (μg/m^3 ^for PM_2.5 _and EC, ppb for NO_2_)

Although the fixed-site EPA monitors combined with our three continuous monitoring sites cannot fully capture spatial heterogeneity, the continuous measurements can provide some additional insight about temporal trends potentially influenced by the DNC. On a weekly average basis, concentrations of PM_2.5 _were significantly lower during the DNC than the average of the weeks before and afterwards (considering identical days of the week) (Figure 6). Mean concentrations ranged from 18–24 μg/m^3 ^across EPA monitors during non-DNC weeks, versus 12–17 μg/m^3 ^during the DNC (30–36% lower). Of note, DustTrak measurements of PM_2.5 _(taken in Cambridge) do not correspond directly with gravimetric measurements [25,26], so these values should not be directly compared with the values from other monitors. The concentration difference between the DNC and non-DNC periods was smaller for nitrogen dioxide, although the differential varied more by site, ranging from no change at the urban high-traffic monitor to a 16% reduction at the urban central city monitor. EC concentrations decreased by 8% at the urban monitor near I-93 but increased by 6% at the urban central city monitor.

Considering the time trends in more detail, for PM_2.5_, there are no discernible time trends corresponding to road closures, nor is there indication of greater concentration reductions at monitors proximate to road closures. In contrast, for elemental carbon, concentration reductions were greater at the monitors close to I-93. In addition, both the EPA monitor near I-93 and our study monitor near I-93 (South Boston) had relatively lower concentrations during the road closure periods than during other times of the day (Figure 6). For nitrogen dioxide, no continuous monitoring sites were proximate to the road closures, but only an urban site strongly influenced by local traffic events did not have a significant decrease in concentrations during the DNC. Reduced NO_2 _concentrations during the DNC could theoretically be related to elevated ozone levels; however, ozone concentrations were actually lower during the DNC (i.e., 62 ppb during non-DNC weekdays vs. 41 ppb during DNC weekdays for 1-hour maxima at Lynn). In general, the differential patterns across pollutants and monitors are to be expected given differences in traffic patterns, diurnal concentration influences, and relative contributions of traffic to ambient concentrations.

## Discussion

Our monitoring study provided some insight into the changes in air pollution within a metropolitan area as a result of a natural experiment resulting in changes in traffic volume and patterns. Analyses of highway traffic data indicated that there were some reductions in traffic volume, although the volume only decreased by a maximum of 30% on a daily average basis and was largely isolated to I-93, especially north of the city. Given these data, it would have been anticipated that we would detect small concentration reductions of traffic-related air pollution proximate to this highway, but small concentration increases near significant exit routes from this highway. Our nitrogen dioxide analysis confirmed these expectations. In addition, we found concentration reductions of elemental carbon that were greater at monitoring sites closer to I-93 and during the times of the road closures. Although PM_2.5 _did show an overall concentration decrease, the temporal pattern for PM_2.5 _did not correspond with the road closures or with patterns for elemental carbon and nitrogen dioxide. The PM_2.5 _differential is likely associated to a greater extent with long-range atmospheric transport differences rather than with local traffic.

Although it appears that we were able to capture the effects of this short-term change in traffic patterns to some degree, the relationships were weak and only detectable in a subset of our analyses, and there are a number of limitations that must be acknowledged. First, our primary comparison is between the week of the DNC and the weeks before and after the DNC. Given the relatively small number of days captured in the analysis, our findings could easily be explained by general meteorological trends that happened to correspond with our sampling periods. There were no obvious patterns from our evaluation of local meteorological or air pollution data, but significant rainfall immediately prior to the DNC could have influenced concentration trends in following days. However, wind speeds and ozone concentrations were both lower during the DNC, factors that would tend to increase near-source traffic contributions to nitrogen dioxide, so the true impact of the road closures may have been greater than the increments we estimated.

In addition, the meteorological variables we evaluated did not capture all aspects of long-range transport, which may have influenced PM_2.5 _concentrations to a significant extent. An alternative analytical approach would have involved a more formal selection of comparison dates from previous years based on defined matching characteristics (i.e., mixing height, wind speed, precipitation, temperature and humidity). While this would have been more rigorous in terms of meteorological factors, this would not have captured spatial patterns of concentration changes (given NO_2 _monitoring data at a small number of sites), and changing traffic flows over time given ongoing construction would make this comparison more complicated to interpret.

In addition, our primary integrated sampling conclusions were based on *a priori *classifications of road segments, but we did not have empirical traffic count data on auxiliary roads that could confirm these expectations (as traffic counts were only available for highways and the single road segment we monitored). In particular, for some of the roads where we hypothesized that traffic volumes would increase, it is impossible to know whether this actually occurred given available data. Traffic count data from I-93 north of Boston did demonstrate diminishing volume closer to Boston (relative to comparison weeks), which provides some evidence of increased exiting on our hypothesized auxiliary roads, but we cannot confirm traffic patterns beyond the exit. Anecdotal evidence (i.e., conversations with residents of Somerville) does not support the hypothesis of traffic volume increases on these local roads. However, it is possible that traffic decreased at these sites, but not as much as it decreased elsewhere, resulting in higher relative concentrations (with absolute levels affected by meteorology and photochemistry).

While we are lacking any formal non-highway traffic data, some ancillary analyses can be used to help determine if there were significant traffic pattern shifts. We estimated normal traffic volume in a 200-meter radius around each of our NO_2 _sampling sites, using average traffic volume data from the Massachusetts Highway Department and a kernel-weighting function which more heavily weights traffic on road segments closer to the monitor. If the traffic composition shifted noticeably from the typical pattern in a given week, we would expect that the correlation between NO_2 _and the average traffic score would be lower, given increased error in the traffic volume term. For the non-DNC weeks, we find that there is a moderate association between mean NO_2 _concentration and traffic (Spearman correlation = 0.23, p = 0.18). During the week of the DNC, this relationship weakens (Spearman correlation = 0.09, p = 0.61). While somewhat speculative, this result supports our original hypothesis that traffic patterns shifted during the DNC.

Our conclusions were also based on measurement of a limited number of pollutants. In particular, we only had the capability of fully evaluating spatial patterns of nitrogen dioxide, which is a strong marker of traffic but may not capture all exposures of interest from a public health perspective. We were also limited in terms of our ability to be proximate to some closed-down roads, given logistics of sample deployment, which may have contributed to detection problems given substantial gradients of some pollutants as a function of distance from the roadway. This could have contributed some systematic biases, as it was easier to locate samplers on surface roads than immediately adjacent to highway segments. That being said, a buffer analysis in ArcGIS indicates that our nitrogen dioxide badges were within 200 meters of the road of interest at all sites where we had a specific roadway of concern, which should reasonably capture the impact of the roadways.

Finally, we were somewhat limited by the fact that the natural experiment did not yield the most extreme hypothesized scenario – massive congestion on selected surface roads and highways with large volume reductions on closed highway segments. The reduction in traffic volume on I-93 was sufficient to avoid noticeable congestion in communities north of Boston, and some of the road closures were not strictly enforced during all time periods given reduced traffic flow and the timing of the DNC. This is an inherent limitation of a field campaign aimed at an event with unknown impacts (in magnitude, direction, or location). The fact that we found some evidence of an effect even under these circumstances indicates that our approach may be useful in future studies.

In general, our findings allow us to draw conclusions both about the potential influence of changes in traffic patterns and about the monitoring strategies that should be employed in future investigations. On the former point, our study illustrated that road closures or other means to limit vehicular traffic in selected areas can influence both the magnitude and distribution of traffic-related air pollution. Studies evaluating such events or policies should carefully evaluate the changes in traffic flow on both major and minor roads and should characterize the resulting spatial air pollution patterns.

On the latter point, our spatial nitrogen dioxide analysis provided useful insight about the influence of shifts in traffic patterns. These passive samples also have the advantage of simplicity, which can be important in a project involving community participants in badge deployment (as ours did). Clearly, a significant downside of this sampling approach is the fact that the anticipated traffic shift did not occur during all sampling hours or during all sampling days. We took one-week average measurements, to ensure concentrations above the limit of detection and interpretable data, as well as to address logistical issues. However, the DNC only lasted for four days, with road closures during a fraction of the day, giving us less power to detect the effect of the road closures. That being said, some of the local traffic impacts likely lasted throughout the week and at other hours of the day (i.e., commuters who left town or shifted to public transportation for the week).

Our continuous monitors could better capture diurnal variability in principle, but given a smaller number of monitoring sites and anticipated spatial heterogeneity, conclusions depended on where the monitors were located. In addition, with this approach, instrument failure can significantly influence overall data quality; two of our three aethalometers did not yield valid measurements throughout the monitoring period, which greatly impaired our ability to draw conclusions about spatial patterns for elemental carbon concentration changes. While this can be ameliorated to some extent, there are many contexts in which the monitors must be deployed quickly and cannot be maintained extensively during the study, especially in a community-based research setting.

Future studies targeting specific traffic events or other significant short-term changes in emission patterns should employ integrated sampling at numerous locations, to yield insight about locations of concern as well as the effects of the emission changes. We additionally recommend that future investigations of this sort gather more detailed information about traffic volumes on non-highway roads of interest, with visual traffic counts by community members potentially supplementing automatic traffic counts. We also recommend more extensive spatial coverage, as applicable, to ensure sufficient power to detect the effects of interest, including standardized distances to roadways to the extent possible. Integrated sampling should be conducted with an averaging time that most closely corresponds with the event of interest, to the extent possible, and should be conducted for a long enough time period to establish a record of concentrations pre-change and post-change (and to detect aberrations in time trends). Furthermore, we recommend that these efforts be supplemented by selected continuous monitoring, as the continuous measurements can provide greater insight about time trends and can enhance causal interpretations.

## Conclusion

The Democratic National Convention of 2004 resulted in short-term reductions in traffic volume on a major highway, with some evidence for relative increases in the use of auxiliary roads near exits of this highway. With a spatial sampling protocol using passive nitrogen dioxide badges, there was weak evidence for an average effect on concentrations, but there was evidence that concentrations declined near closed-down roads but increased on auxiliary roads, relative to comparison weeks. Continuous monitoring data for multiple air pollutants demonstrated the spatial heterogeneity in traffic-related air pollution impacts and confirmed expected temporal trends at monitors proximate to road closures. We conclude that sampling campaigns such as this one represent a viable approach for the evaluation of the impact of local emissions changes, but that more detailed source and/or emissions characterization would be warranted to increase the likelihood of detecting the impacts.

## Competing interests

The author(s) declare that they have no competing interests.

## Authors' contributions

JIL drafted the manuscript and conceived of the study. LKB participated in study design, sampling, and statistical analysis of continuous monitoring data. JEC participated in study design, sampling, and GIS analyses. All authors read and approved the final manuscript.
